# Optical spin noise spectra of Rb atomic gas with homogeneous and inhomogeneous broadening

**DOI:** 10.1038/s41598-017-08759-4

**Published:** 2017-08-31

**Authors:** Jian Ma, Ping Shi, Xuan Qian, Yaxuan Shang, Yang Ji

**Affiliations:** 1 SKLSM, Institute of Semiconductors, Chinese Academy of Sciences, Beijing, 100083 People’s Republic of China; 2 College of Materials Science and Opto-Electronic Technology, University of Chinese Academy of Sciences, Beijing, 100049 People’s Republic of China

## Abstract

We study the optical spin noise spectra of Rb atomic gas with different broadening mechanisms. The first is homogeneous broadening using 250 Torr nitrogen buffer gas, while the other mechanism is inhomogeneous broadening via the Doppler effect without buffer gas. Spin noise signals are measured by the typical spin noise spectroscopy geometry (single-pass geometry) and the saturated absorption spectroscopy geometry (double-pass geometry). In the homogeneously broadened system, the line shape of the optical spin noise spectra shows a pronounced dip that vanishes at the center of the band in both geometries. In the inhomogeneously broadened system, however, a peak in the single-pass geometry and a dip in the double-pass geometry at the band center are observed. The difference between the optical spin noise spectra from these two systems arises from their different level-broadening mechanisms.

## Introduction

Spin-based electronic systems have been attracting more and more attention in recent years, since a spin can carry quantum information and thus is a promising candidate for new information technologies^1, 2^. Optical methods, such as Hanle experiments and time-resolved Faraday/Kerr rotation spectroscopy, play important roles in spintronics research. However, these methods employ optical excitation to create a preferential spin orientation, which demolishes the intrinsic spin dynamic^3^. This disadvantage can be avoided in all-optical spin noise spectroscopy (SNS), a new kind of Faraday rotation (FR) experiment. The SNS method uses a linearly polarized, below-band-gap laser as the probe light to sense the FR of the system, that is, the projection of the average spin polarization along the direction of light propagation, which fluctuates even under thermal equilibrium^4^.

SNS is an elegant method to study spin dynamics at thermal equilibrium without extra optical excitation. Crooker *et al*. performed the first measurements in atom optics in 2004 and predicted that SNS could be used for semiconductors^5^. In 2005, Oestreich and co-workers conducted SNS measurements of a semiconductor system^6^. Since then, this technique has been used to measure the Land*é* factor, nuclear spin, isotope abundance ratios, hyperfine splittings, spin coherence lifetimes of alkali-metal atoms^5, 7, 8^ and the properties of electrons in semiconductors^6, 9–12^. A breakthrough in this field was achieved when the sweeping spectrum analyzer was replaced by a data acquisition card with a real-time fast Fourier transform (FFT) processing system^3, 13^, which enabled a much higher signal-noise ratio, leading to spin detection in quantum dot ensembles^13–16^. The spin noise spectrum could further be enhanced by a Fabry-Perot cavity allowing multiple passes of the light through the magneto-optical medium^17–19^.

Experimental and theoretical studies of SNS usually focus on the off-resonant probing regime. However, spin noise spectra can also be obtained under resonant and quasi-resonant conditions in atomic gas. While the SNS in such regimes does perturb the system, it also provides more information about the system. Crooker *et al*. studied the SNS in natural rubidium atomic gas, and showed that the integrated spin noise became larger when the laser frequency was closer to the center frequency at the off-resonant probing regime^5^. Oestreich *et al*. performed SNS measurements under resonant as well as non-resonant probing conditions in rubidium atomic gas, and clear signatures of the coherent coupling were observed for the participating electronic levels^7^. Zapasskii and co-workers predicted that the total spin noise power as a function of the probe light frequency depended on whether the system was homogeneously or inhomogeneously broadened^20^, which was followed by experimental verification from SNS measurements of two different materials. The first material was the homogeneously broadened D1 line of potassium atoms and the other was the inhomogeneously broadened band of the InGaAs/GaAs quantum dots^21^. In what follows, the spin noise amplitude as a function of the probe light frequency will be referred to as optical spin noise (OSN) spectra.

Here we report the OSN spectra of the same material with different broadening mechanisms. The level broadening of rubidium atomic gas can be switched from inhomogeneous broadening to homogeneous broadening by the addition of buffer gas into the vapor cell. The spin noise spectrum as a function of the laser frequency detuning was measured under both the typical SNS geometry (single-pass geometry) and the saturated absorption spectroscopy geometry (double-pass geometry). Our results, especially those measured with a double-pass geometry, clearly demonstrate that the difference of the OSN spectra between the homogeneously and inhomogeneously broadened systems arises from their different level-broadening mechanisms.

## Results

### Experimental setup

The basic idea of SNS is to map the random fluctuations of spins in the sample on the polarization of the probing light, which was a linearly polarized laser transmitted through the sample. Even at thermal equilibrium, the atom ensemble showed stochastic spin polarization at a given time, which imparted FR fluctuations on the probe beam through the spin-dependent indices of refraction for the right- and left-circularly polarized light. These variations of the FR were converted to voltage signals through the combination of a Wollaston prism and a balanced photodiode bridge. The electrical signal from the balanced photoreceiver was continuously collected, computed and accumulated in real time by a field programmable gate array (FPGA)-based data acquisition card (DAC) and finally sent to a computer.

**Figure 1:**
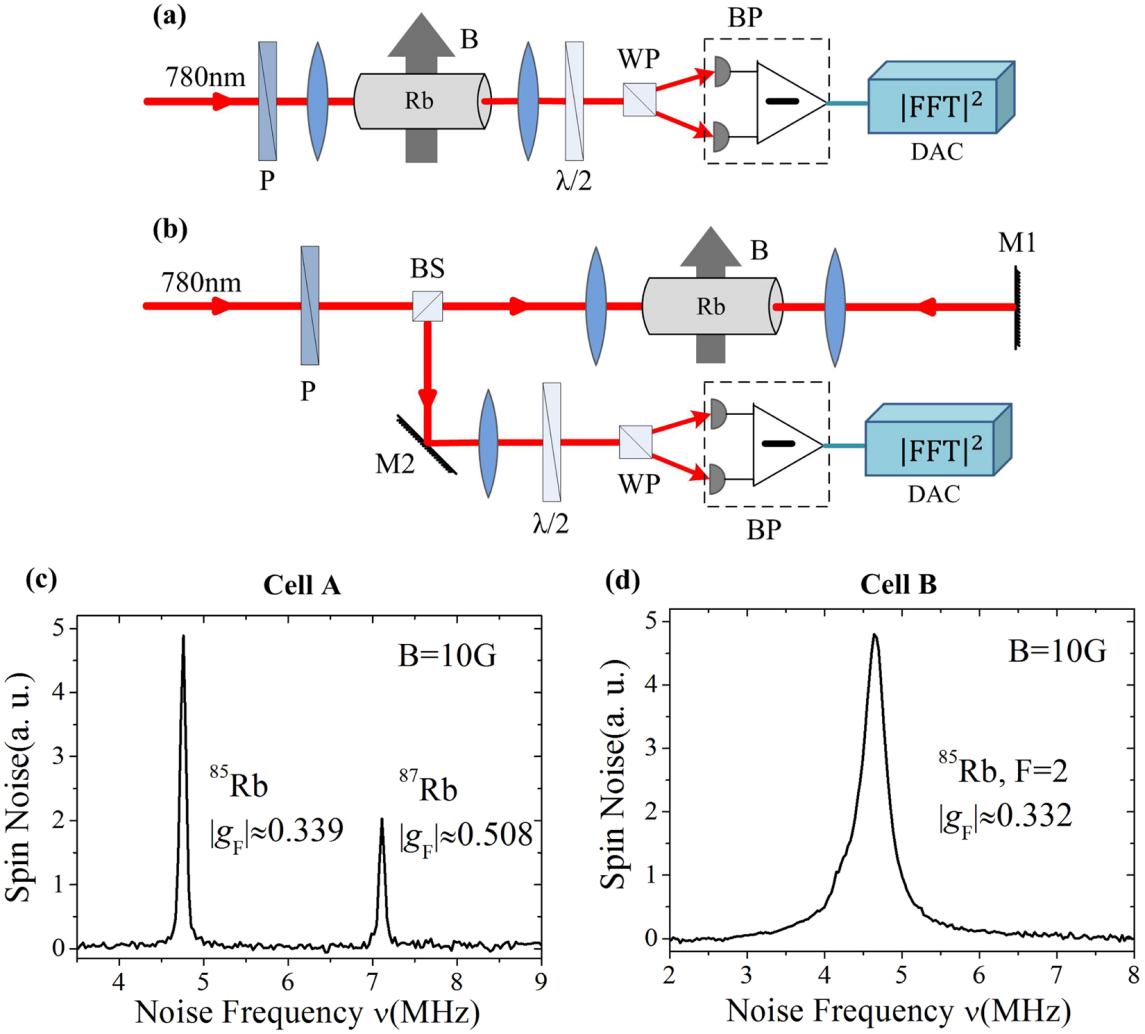
Stochastic fluctuations of the Faraday rotation signal is detected in real time by a balanced photodiode bridge and an FPGA-based DAC. P-polarizer, BS-beam splitter, *λ*/2-halfwave plate, WP-Wollaston prism, BP-balanced photodiodes, M1, M2-mirrors, DAC-data acquisition card. (**a**) Typical experimental schematic of spin noise spectroscopy (single-pass geometry). (**b**) Spin noise spectroscopy under saturated absorption spectroscopy geometry (double-pass geometry). (**c**) Spin noise spectrum in natural rubidium with 250 Torr nitrogen. *T* = 313 K. The laser was detuned Ω_*D*2_ = 7 GHz from the D2 transition (5^2^ *S*_1/2_ to 5^2^ *P*_3/2_, 780 nm) and a laser power *I* = 300 *μ*W. The applied magnetic field *B* = 10 G. (**d**) Spin noise spectrum in natural rubidium without buffer gas, with *T* = 293 K. The laser is detuned Ω_*D*2_ = 0.5 GHz from the D2 transition (5^2^ *S*_1/2_(F = 2) to 5^2^ *P*_3/2_, 780 nm) and the laser power *I* = 300 *μ*W. The applied magnetic field *B* = 10 G.

Two kinds of experimental configurations were used to explore the OSN spectra of Rb atomic gas with homogeneous and inhomogeneous broadening. One was the typical SNS geometry, where the probe beam passes through the vapor cell only once (single-pass geometry, Fig. 1(a)) and the other was the saturated absorption spectroscopy (SAS) geometry, where the probe beam passes through the vapor cell and then backtracks to the beam splitter (double-pass geometry, Fig. 1(b)) so that the rubidium vapor in the cell interacts with two counter-propagating laser beams^22^. The second configuration can avoid the Doppler effect around the band center in the atomic gas, which is the mechanism for the inhomogeneous broadening in the pure Rb gas.

**Figure 2:**
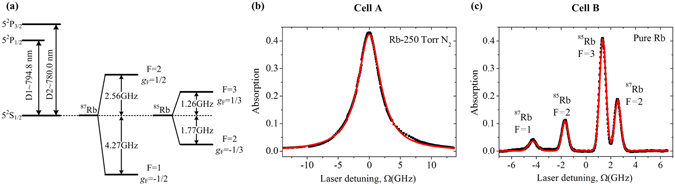
The absorption spectra of rubidium. (**a**) D-line transition and ground-state hyperfine structure of rubidium^28, 30^. (**b**) The absorption spectrum of natural rubidium with 250 Torr of nitrogen buffer gas (black squares) and fitting curve (red line) as a function of laser detuning, with *T* = 313 K. (**c**) The absorption spectrum of natural rubidium without buffer gas (black squares) and fitting curve (red line) as a function of laser detuning, with *T* = 293 K.

The linearly polarized probe light was provided by a Ti:sapphire CW tunable laser with a spectral line width of approximately 100 kHz. The light was focused by a lens with a 400 mm focal length to a beam diameter of approximately 100 *μ*m at the center of the rubidium vapor cell. Two 60 mm-long rubidium vapor cells were used in this work, where one contained natural rubidium atomic gas with 250 Torr of N_2_ (cell A), while the other cell contained no buffer gas (cell B). A furnace tuned the temperature of the cell and thus the density of the rubidium gas contained in it. Figure 2(a) shows the D-line transition and ground-state hyperfine structure of rubidium atoms. Figure 2(b,c) depict the optical absorption spectra of cell A and cell B, respectively. The density of the rubidium vapor was approximately 10^11^ *cm* ^−3^ for cell A at *T* = 313 K and approximately 10^10^ *cm* ^−3^ for cell B at *T* = 293 K. The absorption spectrum of cell A showed a feature with the full width at half maximum (FWHM) of 4.4 GHz and could be well-fitted with a Lorentzian curve. Owing to the buffer gas in the system, the spectral line broadening was determined by the collision between rubidium atoms and N_2_ molecules, which meant that the system was homogeneously broadened^23^. In Fig. 2(c), the two peaks in the center of the spectrum arose from the isotope ^85^Rb while the outer lobes were attributed to ^87^Rb. These features came in pairs because of the hyperfine interaction and hence were split in the 5^2^ *S*_1/2_ ground-state. A Gaussian fit was used to verify the absorption spectrum, and the FWHM of the four peaks were in the range of 400~600 MHz, meaning that the line broadening of the absorption spectrum mainly arose from the Doppler effect (i.e., level shift arising from the thermal velocity of the atoms), and the system was inhomogeneously broadened.

A 80 MHz balanced bridge with 20 V/mW peak conversion gain (New Focus 1807) was used to convert FR fluctuations to voltage signals, which limited the detection band width to 80 MHz. The electrical signal was collected and analyzed by a FPGA-based DAC similar to that described by Crooker *et al*.^13^. The FPGA-based DAC had a sampling rate of 1 GS/s and its input range was −0.5 V~+0.5 V with 8-bit resolution. An electrical band-pass filter with 0.5 MHz low cut-off frequency was used before the DAC. The FPGA was able to process fast-Fourier transform (FFT) algorithms at 32 k-points in real time while the DAC continuously read the data, which means the experimental frequency resolution was 31.25 kHz. Before the results were sent to a computer, 10,000 power spectra (|FFT|^2^) were accumulated. The FPGA-based DAC exhibited a typical data usage ratio of 50%, which could further be improved by increasing the data transmission speed between the DAC and the computer^24^.

A magnetic field transverse to the light propagation direction shifted the peak of the spin noise spectrum from zero frequency to the Larmor precession frequency *ν*_*L*_ = *gμ*_*B*_ *B*/*h*, where *g* is the Land*é* factor, *μ*_*B*_ is the Bohr magneton, *B* is the external magnetic field and *h* is the Planck constant. The noise background arising from the optical fluctuation of the laser and electrical noise of the balanced receiver were eliminated by subtracting the spin noise spectra measured with a background field from that measured with the target magnetic field (10 G). Here we chose the background field to be 50 G in order to avoid the effect of the terrestrial magnetic field^7^. Figure 1(c,d) show typical spin noise spectra that were acquired from cell A and cell B after 7.5 minutes of accumulation. The peak position and the FWHM of the spin noise spectrum indicated the absolute value of the effective *g*-factors and the spin-dephasing time of rubidium atoms, respectively. The FWHM of the spin noise spectrum in Fig. 1(c,d) was 70 kHz (400 kHz), indicating that the spin-dephasing time of rubidium atomic gas with 250 Torr N_2_ (without) buffer gas was approximately 14 *μ*s (2.5 *μ*s). This was much lower than the known rubidium spin lifetime (~100 ms)^25^, largely as a result of the resolution of the FPGA-based DAC (cell A)^26^ and the transit time of the atoms across the light beam (cell B)^7^.

### Experimental results

**Figure 3:**
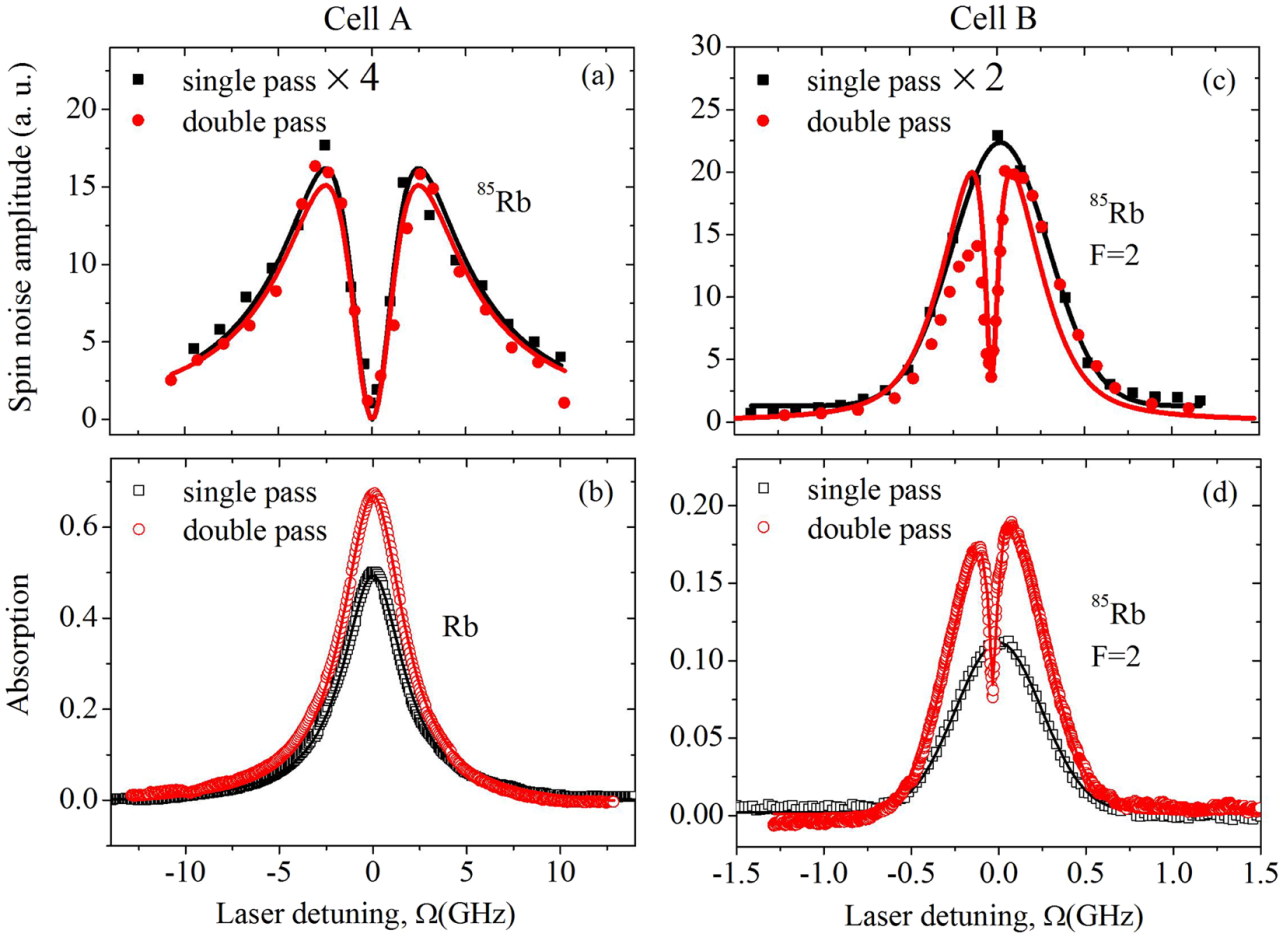
OSN spectra of Rb atomic gas with single-pass and double-pass geometry. (**a**,**b**) The OSN spectra and the absorption spectra of ^85^Rb in cell A at temperature *T* = 313 K. (**c**,**d**) The OSN spectra and the absorption spectra of ^85^Rb (5^2^S_1/2_(F = 2) to 5^2^P_3/2_) in cell B at *T* = 293 K, with the transverse magnetic field *B* = 10 G, and the laser power *I* = 300 *μ*W for single-pass geometry and *I* = 1 mW for double-pass geometry. To enable a direct comparison of the spectra, the OSN spectra measured by single-pass geometry is multiplied by a factor of 4 and 2 for cell A and cell B, respectively.

We measured the spin noise spectra as a function of laser frequency detuning in two rubidium vapor cells, where cell A contained a buffer gas and cell B did not. Figure 3 shows the OSN spectra and the absorption spectra measured with a single-pass and double-pass geometry in the homogeneously and inhomogeneously broadened systems. It should be noted that we have only shown the experimental results of ^85^Rb in cell A and ^85^Rb (5^2^S_1/2_(F = 2) to 5^2^P_3/2_) in cell B, as the rest of the experimental data were similar. Here we use the spin noise amplitude instead of use the integrated spin noise because the FWHM of the spin noise spectra varies as laser detuning changes^21^, which would otherwise make our model too complicated (see below).

In the single-pass geometry, while the absorption spectra of both systems exhibited similar peaks (although with quite different FWHM), the amplitude of spin noise spectra at different detuning of the probe laser in the two systems was drastically different. The line shape of the OSN spectrum showed a pronounced dip and vanished at the band center in the case of the homogeneously broadened system (cell A), whereas the inhomogeneously broadened system only exhibited a peak at the band center (cell B). Our experimental results were consistent with previous studies^20, 21, 27^.

In the double-pass geometry, the absorption spectrum of ^85^Rb in cell B exhibited a sharp dip with a width of 63 MHz (the famous Lamb dip^22^), as the laser frequency approached the atomic transition frequency. The Lamb dip can be understood by the thermal atoms having a Maxwell-Boltzmann distribution of the velocity centred at the velocity *u* = 0. Owing to the Doppler effect, the counter-propagating laser beams interacted with the same atoms at *u* = 0. The depletion of the ground state atoms caused by the forth-going beam then led to a reduction in the absorption of the back-coming beam. In other cases (where *u* ≠ 0), the counter-propagating laser beams interacted with different atoms. Hence, the laser burned a “hole” in the middle of the ground-state distribution^22^. The asymmetrical absorption profile in Fig. 3(d) arose from the difference of the hyperfine transition strength factors^28^. The “hole” did not appear in cell A (Fig. 3(b)) as it was collision broadened.

In the homogeneously broadened system (cell A), the line shape of OSN spectra measured with double-pass geometry was the same as that measured with single-pass geometry (Fig. 3(a)). However, the OSN spectra profile of ^85^Rb in cell B, as shown in Fig. 3(c), was quite different from that obtained with a single-pass geometry, where a dip appeared around the center. This was quite similar to the Lamb dip, however, its minimum was much smaller. The widths of the inhomogeneous broadening seemed to be different in the double-pass and the single-pass geometries. At the same time, the line shape of Fig. 3(c) was even more asymmetrical than that in Fig. 3(d). We ascribed these to the asymmetry of the absorption profile, since the spin noise signal was proportional to *I* ^2^, while the absorption signal was proportional to *I*, where *I* is the intensity of the transmitted light.

## Discussion

Next, we will explain the experimental results from the interaction between light and atoms, which may be described as $L({\rm{\Omega }}-{{\rm{\Omega }}}_{0})=\tfrac{\gamma }{{({\rm{\Omega }}-{{\rm{\Omega }}}_{0})}^{2}+{\gamma }^{2}}$, where Ω is the probe light frequency, Ω_0_ and *γ* are the central frequency and the natural line width of a two-level system, respectively. If the atoms population on the energy level is *N*, the statistical spin polarization in thermal equilibrium at any time is approximately $\sqrt{N}$ ^5^. The FR angle *δθ* of a single level can be computed as refs 21 and 27

1 $$\delta \theta ({\rm{\Omega }})\propto \sqrt{N}\frac{{\rm{\Omega }}-{{\rm{\Omega }}}_{0}}{{({\rm{\Omega }}-{{\rm{\Omega }}}_{0})}^{2}+{\gamma }^{2}}.$$

The spin noise signal was proportional to the squared FR spectrum

2 $$S({\rm{\Omega }})\propto {[\delta \theta ({\rm{\Omega }})]}^{2},$$

The spin noise signal approached zero since the FR became zero at the center of the band.

The sum of the optical spectra of each atom composed the optical spectrum of the system. In cell A, owing to the collisions between rubidium atoms and N_2_ molecules, the line width of all the rubidium atoms in the vapor cell was homogeneously broadened to 4.4 GHz^29^. Hence, all of the optical spectra of the atoms were identical. The line shape of the absorption and the FR spectra of the system were same as those of an individual atom. Hence, the OSN spectrum in the single-pass geometry is

3 $${S}_{h1}({\rm{\Omega }})\propto {[\delta \theta ({\rm{\Omega }})]}^{2},$$

and a dip appeared near the center of the band. The fitting curve is shown in Fig. 3(a) (black line) with *γ* = 4.4 GHz, which equals the FWHM of the absorption spectrum.

In the double-pass geometry, the back and forth travel time of the light was much less than the spin lifetime and the transit time of the atoms. Therefore, the counter-propagating laser beams interacted with the same atoms. Since the probe light passed through the vapor cell twice, the FR angle in this geometry is 2*δθ*. Hence, the OSN spectrum in the double-pass geometry is

4 $${S}_{h2}({\rm{\Omega }})\propto {[2\delta \theta ({\rm{\Omega }})]}^{2}=4{S}_{h1}({\rm{\Omega }}).$$

The fitting curve is shown in Fig. 3(a) (red line) with *γ* = 4.4 GHz, which equals the FWHM of the absorption spectrum. The predict result was the spin noise signal measured with double-pass geometry will be four times that with the single-pass geometry and the experimental results agreed well with the predicted values.

In cell B, the system was inhomogeneously broadened owing to the Doppler effect. Every individual velocity class of atoms exhibited sharp optical transitions as narrow as several tens of MHz, but the distribution of atomic velocities led to a broad inhomogeneous line width of approximately 490 MHz in the ensemble measurements^29^.

For a given velocity class subsystem, the line width was homogeneously broadened and the line shape of the OSN spectrum could be described by Eq. (2). In the inhomogeneously broadened system, the OSN spectra consisted of a huge number of subsystems with different velocity classes. Under single-pass geometry, the line shape of the OSN spectrum can be computed as

5 $${S}_{i1}({\rm{\Omega }})\propto \int {\{\frac{{\rm{\Omega }}-{{\rm{\Omega }}}_{0}(1+\tfrac{u}{c})}{{[{\rm{\Omega }}-{{\rm{\Omega }}}_{0}(1+\tfrac{u}{c})]}^{2}+{\gamma }^{2}}\}}^{2}\,\exp \,(-{u}^{2}/{{\rm{\Gamma }}}^{2})\,{\rm{d}}u,$$

where *u* is the velocity of the atoms, *c* is the light velocity, *γ* is the natural line width of a two-level system and Γ is the FWHM of the Gaussian profile of the absorption spectrum in the inhomogeneously broadened system. The fitting curve is shown in Fig. 3(c) (black line) with Γ = 490 MHz, which equals the FWHM of the absorption spectrum, and *γ* = 63 MHz, which equals the FWHM of the Lamb dip in Fig. 3(d). Although the OSN spectrum of every velocity class atoms was similar for the homogeneously broadened systems, the absorption spectrum of the system was Gaussian shape, which ultimately determined the line shape of the OSN spectrum in the inhomogeneously broadened system. The fitting curve was mainly determined by the inhomogeneous width Γ, and it was not sensitive to homogeneous width *γ*, as long as *γ* was much less than Γ^21^.

In a double-pass geometry, the Doppler effect could be avoided and the two counter-propagating laser beams interacted with the same class of atoms when the probe light frequency was around the band center, so the FR angles of the back and forth travelled light were correlated. Hence, we have measured the OSN spectrum of atoms in the range of the natural line width around the central frequency in the inhomogeneously broadened system. This was the reason a dip around the central frequency appeared in Fig. 3(c) (red circles). Note that the Doppler effect could only be avoided at the regime where the probe light frequency was around the band center. When the probe light was far from the central frequency, the two counter-propagating laser beams interacted with different classes of atoms with different velocities, thus the FR angles of the back and forth travelled light were uncorrelated. Hence, the OSN spectrum is

6 $$\begin{array}{lll}{S}_{i2}({\rm{\Omega }}) & \propto  & \int {\{\tfrac{{\rm{\Omega }}-{{\rm{\Omega }}}_{0}(1+\tfrac{u}{c})}{{[{\rm{\Omega }}-{{\rm{\Omega }}}_{0}(1+\tfrac{u}{c})]}^{2}+{\gamma }^{2}}+\tfrac{{\rm{\Omega }}-{{\rm{\Omega }}}_{0}(1-\tfrac{u}{c})}{{[{\rm{\Omega }}-{{\rm{\Omega }}}_{0}(1-\tfrac{u}{c})]}^{2}+{\gamma }^{2}}\}}^{2}\,\exp \,(-{u}^{2}/{{\rm{\Gamma }}}^{2})\,{\rm{d}}u\\  & \approx  & 2{S}_{i1}({\rm{\Omega }})\,[1-\alpha A({\rm{\Omega }})],\end{array}$$

where $A({\rm{\Omega }})=\tfrac{{\gamma }^{2}}{{({\rm{\Omega }}-{{\rm{\Omega }}}_{0})}^{2}+{\gamma }^{2}}$ is the correlation coefficient. The fitting curve is shown in Fig. 3(c) (red line) with the same Γ = 490 MHz and *γ* = 63 MHz as those for Eq. (5). In the region where the probe light frequency was far from the center frequency, the correlation coefficient equalled zero and the relationship of the OSN spectra in the inhomogeneously broadened system was *S*_*i*2_(Ω) = 2*S*_*i*1_(Ω). The experimental results are show in Fig. 3(c), which showed a good agreement between the signals measured in the two different geometries. The phenomenological parameter *α* was introduced to take into account the absorption of the sample since it decreased the intensity of the back-coming light compared with the forth-going light (more details are in the supplementary information). For the data-fitting process in Fig. 3(c), we used *α* = 0.8, that is, *S*_*i*2_(Ω) ≈ 2*S*_*i*1_(Ω) [1 − 0.8*A*(Ω)]. We made several measurements with *I* = 0.5, 1.0, 1.5 mW and all the results were similar (see data in the supplementary information), with the dip being approximately 20% of the peak value as that shown in Fig. 3(c).

In summary, we have performed spin noise spectra in the same material with different broadening mechanisms. Rb atomic gas with and without nitrogen buffer gas were used to represent the homogeneously and inhomogeneously broadened systems, respectively. Spin noise signals were measured with single- and double-pass geometry. The line shape of the OSN spectrum in the single-pass geometry exhibited a pronounced dip that vanished at the band center in the homogeneously broadened system, whereas it was a peak in the inhomogeneously broadened system. These observations agreed well with the former work by Zapasskii *et al*.^21^. In the double-pass geometry, the line shape of the OSN spectrum in the homogeneously broadened system was the same as that in the single-pass geometry. However, the line shape of the OSN spectrum in the inhomogeneously broadened system showed a Lamb dip-like line shape in the double-pass geometry, in contrast to the peak in the single-pass geometry. Our results, especially those of the inhomogeneously broadened system measured with the double-pass geometry, clearly show that the difference of spin noise signals between these two systems arises from their different level-broadening mechanisms.

## Electronic supplementary material

Supplementary Information
