# Supplementary material for: Optical spin noise spectra of Rb atomic gas with homogeneous and inhomogeneous broadening
Source: Sci Rep. 2017 Aug 31;7:10238. doi: 10.1038/s41598-017-08759-4 (PMC5579247; doi:10.1038/s41598-017-08759-4)
Supplement: Supplementary file 1 — Supplementary Information [file 41598_2017_8759_MOESM1_ESM.pdf]

SUPPLEMENTARY INFORMATION FOR
Optical spin noise spectra of Rb atomic gas with homogenous and inhomogeneous broadening

Jian Ma[1,2], Ping Shi[1,2], Xuan Qian[1], Yaxuan Shang[1,2], and Yang Ji[1,2]
[1]*SKLSM, Institute of Semiconductors, Chinese Academy of Sciences, Beijing 100083, People's Republic of China*
[2]*College of Materials Science and Opto-Electronic Technology,*
University of Chinese Academy of Sciences, Beijing 100049, People's Republic of China
[*]*Correspondence and requests for materials should be addressed to Y.J. (jiyang@semi.ac.cn)*

DETAILS OF THE NORMALIZATION WITH LASER POWER

The detected FR signals by the DAC show $F' \propto (I \cdot \delta\theta)$, where I is the transmitted light power and $\delta\theta$ is the FR angle. The relationship between the spin noise signal and FR signal detected by the DAC is $S' \propto F'^2$ since the data from the FPGA-based DAC is Fourier spectra squared. Hence, $S' \propto (I \cdot \delta\theta)^2$. In the single-pass geometry, the probe beam passes through the vapor cell only once and the spin noise signal can be computed as $S'_1 \propto (I_0 T_{cell} T \delta\theta)^2$ in both the homogeneously and inhomogeneously broadened systems, where I_0 is the incidence light power, $T_{cell} \approx 0.7$ is the optical transmission of the cell, T is the optical transmission of the Rb atom gas that can be derived from Figure 3. In the double-pass geometry, according to Figure 1, the intensity of the probe light can be computed as $I = (1/2)I_0(T_{cell}T)^2R^2$, where R=0.95 is the reflectivity of the mirrors M1 and M2 in Figure 1(b), the factor 1/2 is a result of the beam splitter. For homogeneously broadened system, the two counter-propagating laser beams interact with the same atoms and the FR angle of the back and forth travelled light are correlated. Thus, the OSN spectra shows $S'_{h2} \propto (I \cdot 2\delta\theta)^2 = 4S'_{h1}$. For the inhomogeneously broadened system, when the probe light frequency is far from the centre frequency, the two counter-propagating laser beams interact with different classes of atoms with different velocities and the FR angles of the back and forth travelled light are uncorrelated, the OSN spectra shows $S'_{i2} \propto I^2 \cdot 2(\delta\theta)^2 = 2S'_{i1}$.

The relationship between the detected spin noise signal S' by the DAC and the spin noise signal S is $S = S'/I^2$.

THE DEPTH OF THE DIP WITH DIFFERENT PROBE-LASER INTENSITIES

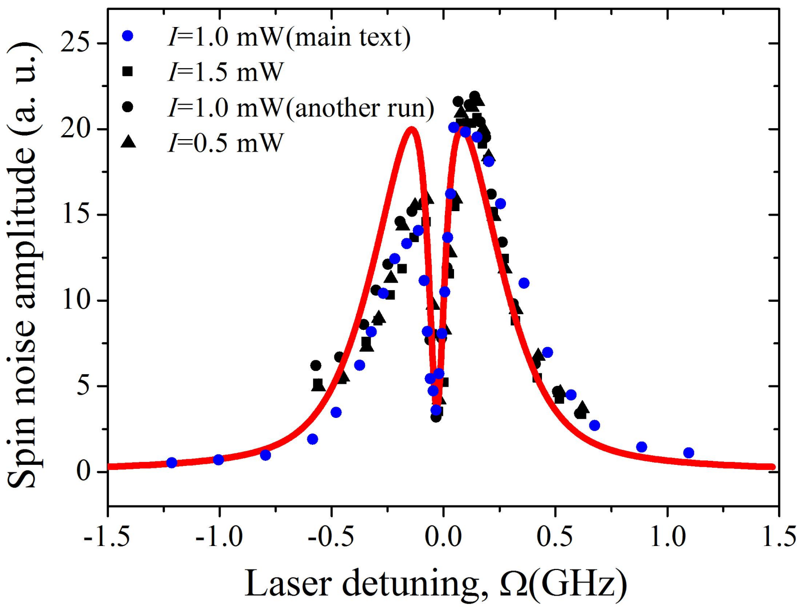

FIG. 1: Spin noise spectra of ^{85}Rb ($5^2S_{1/2}$(F = 2) to $5^2P_{3/2}$) at different detuning of the probe laser in cell B with a double-pass geometry. The transverse magnetic field B=10 G. The experimental data are normalized with the laser power.

Actually, Eq. (6) should be written as

$$S_{i2}(\Omega) \propto \frac{1}{I^2(\Omega)} \int \{I_1(\Omega)\frac{\Omega - \Omega_0(1+\frac{u}{c})}{[\Omega - \Omega_0(1+\frac{u}{c})]^2 + \gamma^2} + I_2(\Omega)\frac{\Omega - \Omega_0(1-\frac{u}{c})}{[\Omega - \Omega_0(1-\frac{u}{c})]^2 + \gamma^2}\}^2 \exp(-u^2/\Gamma^2)\mathrm{d}u,$$

where I is the probe light intensity, I_1 and I_2 are the forth-going and back-coming light intensities and $I_1 > I_2$ owing to the absorption of the sample. The dip of the line shape should not approach zero owing to the difference of I_1 and I_2. For simplification, instead of considering the different weights of the back and forth travelled light in Eq. 6, we introduce a phenomenological parameter α to represent it. However, we include this effect into the phenomenological factor α in the main text. The above figure shows the spin noise power of ^{85}Rb at different detuning of the probe laser in cell B with the double-pass geometry. The I=1.0 mW (main text) trace means the data shown in the main text, while the I=1.0 mW (another run) trace means data measured in another run, together with 0.5 mW and 1.5 mW data. We have made several measurements with the probe laser intensity of I=0.5, 1.0, 1.5 mW and all the results are similar.
